# Sequence-based prediction of protein-protein interactions by means of codon usage

**DOI:** 10.1186/gb-2008-9-5-r87

**Published:** 2008-05-23

**Authors:** Hamed Shateri Najafabadi, Reza Salavati

**Affiliations:** 1 Institute of Parasitology, McGill University, Lakeshore Road, Ste. Anne de Bellevue, Montreal, Quebec H9X 3V9, Canada; 2 McGill Centre for Bioinformatics, McGill University, University Street, Montreal, Quebec H3A 2B4, Canada; 3 Department of Biochemistry, McGill University, Promenade Sir William Osler, Montreal, Quebec H3G 1Y6, Canada

## Abstract

We introduce a novel approach to predict interaction of two proteins solely by analyzing their coding sequences. We found that similarity in codon usage is a strong predictor of protein-protein interactions and, for high specificity values, is as sensitive as the most powerful current prediction methods. Furthermore, combining codon usage with other predictors results in a 75% increase in sensitivity at a precision of 50%, compared to prediction without considering codon usage.

## Abstract 2

A new approach based on similarity in codon usage is used to predict protein-protein interactions.

## Background

The need to transform the growing amount of biological information into knowledge has involved several disciplines that, by means of experimental and computational approaches, aim to decipher functional linkages and interactions between proteins [1,2]. Current computational methods for predicting protein-protein interactions demand data that, compared to the huge amount of available genomic sequences, are scarce. Only in a few organisms have features such as essentiality, biological function and mRNA co-expression of genes been partially determined. Also, a combination of different homology-based predictors, including phylogenetic profiles [3], Rosetta stone [4] and interolog mapping [5], has provided incomplete information about interactions of only one-third of all *Saccharomyces cerevisiae *proteins. Hence, a method to identify protein-protein interactions solely on the basis of gene sequences would significantly expand the ability to predict interaction networks.

A few studies have been performed on the prediction of protein-protein interactions based only on amino acid sequence information [6-8]. However, the highest specificity reported in these studies is 86%. Considering the number of possible protein pairs in a genome consisting of no more than 6,000 protein-coding genes, this level of specificity results in the unacceptable number of 2.5 × 10^6 ^false positives. These studies consider protein sequences, and ignore the plethora of information that exists in their coding sequences. The still-unsatisfied demand for reliable sequence-based prediction of protein-protein interactions encourages exploration of relevant sequence features in the genome instead of the proteome.

It has been widely acknowledged that codon usage is correlated with expression level [9]. In addition, it has been shown that codon usage is structured along the genome [10], with near neighbor genes having similar codon compositions. Some function-specific codon preferences have also been hypothesized based on selective charging of tRNA isoacceptors [11] and have been confirmed experimentally [12]. Based on these premises and considering that similarity in mRNA expression pattern and biological function, along with physical gene proximity, are powerful predictors of protein-protein interactions [13], codon usage can be considered as a potential candidate for analysis. The coevolution of codon usage of functionally linked genes has been explicitly reported before [14,15]. These studies suggest that the codon adaptation index (CAI) [16] of functionally related proteins changes in a coordinated fashion over different unicellular organisms. However, identification of this coordination between two genes needs the presence of orthologues in several organisms; hence, many species-specific genes, which are usually the hot spots of attraction for biologists, are excluded. Also, there are genes with very low variation in the CAI over different organisms [14], for which this kind of analysis is unreliable.

In this paper, we show that codon usage of functionally and/or physically linked proteins in an organism contain enough information to enable us to detect these proteins, even in the absence of homologues in other organisms. Furthermore, we show that our method is several times more sensitive than tracking the coordinated changes of codon usage over different organisms, and in fact is one of the best methods for identification of protein-protein interactions.

## Results and discussion

Here we consider three different organisms: *S. cerevisiae*, *Escherichia coli *and *Plasmodium falciparum*. *S. cerevisiae *is a eukaryote with moderate coding G+C content (39.77%), while the genome of *P. falciparum *has an extremely low coding G+C content (23.8%), and *E. coli *is a prokaryote with moderate coding G+C content (52.35%). For each organism, a positive and a negative gold standard set of protein pairs were defined, where a positive gold standard set comprises open reading frame (ORF) pairs that, based on previous reports, encode proteins that interact with each other (either as members of the same protein complex or as functionally linked proteins), and a negative set consists of ORF pairs whose products do not interact with each other (Table 1). It should be noted that the highest resolution of our gold standard positive datasets is the protein complex. Given each ORF pair, we calculated for each codon the value:

**Table 1:** Gold standard sets

Organism	GSTD	References	No. of ORFs	No. of ORF pairs	Comments/details
*S. cerevisiae*	P	[13, 22]	732	3,400	Derived from MIPS [42] complex catalog. We excluded ribosomal proteins to avoid bias towards extreme codon usage similarity of their genes
	N	[13, 22]	2,760	1,442,691	Pairs of proteins that are not localized in the same cell compartment. We excluded ribosomal proteins
					
*P. falciparum*	P	[43]	352	7,689	Protein pairs within the same KEGG [19] pathway
	N	[43]	354	27,367	Protein pairs with KEGG information, excluding pairs in the gold standard positive set
					
*E. coli*	P	[44]	2,196	7,063	Pull-down assay using a His-tagged ORF library
	N	-	3,703	4,437,833	We compiled a set of protein pairs that are not in the gold standard positive set, given that at least one protein from each pair is copurified with an associate protein by Arifuzzaman *et al*. [44]

Each set comprises only ORFs that could be associated with their genomic sequences using the names that were provided in the original references. Self interactions were considered in neither the training nor the testing process. GSTD, gold standard dataset; N, negative; P, positive.

*d*_*ij*_(*c*) = |*f*_*i*_(*c*) - *f*_*j*_(*c*)|

where *f*_*i*_(*c*) and *f*_*j*_(*c*) are relative frequencies of codon *c *in ORF *i *and ORF *j*, respectively (Σ_*k*_*f*_*i*_(*c*_*k*_) = 1 and Σ_*k*_*f*_*j*_(*c*_*k*_) = 1; *k *= 1,2,..64 indicates all 64 codons). Therefore, *d*_*ij *_demonstrates the distance of two ORFs in terms of usage of codon *c*. We found that for almost all codons, distribution of *d *differed between positive and negative gold standard sets (Additional data file 1). Generally, distribution of *d *shifts to smaller values for ORFs within the gold standard positive set, indicating that interacting ORFs are more similar in codon usage profile than non-interacting ORFs. However, this shift is marginal for each codon individually, which means that single codons are weak predictors of protein-protein interactions.

We divided the distribution of *d *for each codon into 50 intervals, for each of which we calculated the likelihood ratio, that is, the fraction of positive gold standards occurring in that interval divided by the fraction of negatives occurring in that interval. Since the mutual information of *d *for each pair of codons was negligible, we combined these likelihood ratios using a naïve Bayes approach (see Additional data files 2 and 3 for a graphical representation). Although obviously not all features were independent from each other (with statistical tests suggesting 10 to 16 independent components; see Additional data file 4), we found that a naïve Bayesian network is more effective than a Bayesian network in which each variable node has one other parent node, perhaps because the increase of the parameters in the latter case causes partial overfitting of the network. Using a tenfold cross-validation method, we evaluated the performance of this naïve Bayesian network in predicting protein-protein interactions. To do so, we divided the gold-standard set into ten random segments; each time we used nine segments as the training set and calculated the combined likelihood ratios for each ORF pair in the remaining segment. We designate the method 'PIC' (for probabilistic-interactome using codon usage).

**Figure 1:**
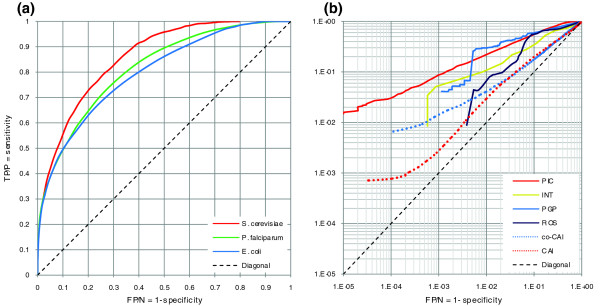
Results of protein-protein interaction prediction by PIC. **(a) **Receiver operating characteristic (ROC) curves of PIC for *S. cerevisiae *(red), *P. falciparum *(green) and *E. coli *(blue). **(b) **Comparison of ROC curves in yeast for PIC (red), interolog mapping (INT, green), phylogenetic profiles (PGP, blue), Rosetta stone (ROS, dark blue), CAI coevolution (co-CAI, blue dotted line) and absolute CAI value (CAI, red dotted line). The dashed line shows the diagonal. The same comparison is shown using the precision-recall curves in Additional data file 10. For interolog mapping, phylogenetic profiles and Rosetta stone, data were retrieved from [41]. FP, false positive; N, negative; P, positive; TP, true positive. Positive and negative test sets are as indicated in Table 1.

Figure 1a summarizes the performance of PIC in *S. cerevisiae*, *P. falciparum *and *E. coli*. For all three organisms, codon usage is a strong predictor of protein-protein interactions. As an extremely G+C poor parasite with a highly biased codon usage [17], the case of *P. falciparum *is of special interest, showing that codon usage is a powerful tool for prediction of interactomes within a wide range of G+C compositions. Figure 1b compares the performance of PIC in yeast with three widely used predictive methods: interolog mapping [5], phylogenetic profiles [3] and Rosetta stone [4,18]. At low rates of false positives, PIC is the most sensitive method, up to seven times more sensitive than the next best method, interolog mapping. Also, for higher rates of false positives, PIC is still more sensitive than interolog mapping and the Rosetta stone approach. Figure 1b also compares PIC with a previous report on identification of protein-protein interactions based on CAI coevolution [14], illustrating up to eight times higher sensitivity for PIC (see Materials and methods for the details of the analysis). Finally, for the sake of comparison, the predictive power of the absolute difference of CAI (see [16] for the definition of CAI and to compare it with PIC) between two genes is investigated, showing a very poor performance (Figure 1b).

**Figure 2:**
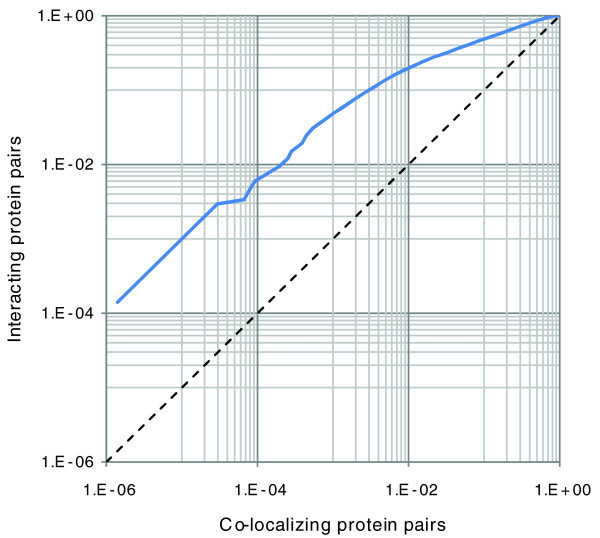
Enrichment of PIC predictions by interacting protein pairs versus protein pairs that co-localize. The horizontal axis shows the fraction of co-localizing protein pairs that match PIC predictions, and the vertical axis shows the fraction of the gold standard interacting protein pairs that match PIC predictions. Rapid enrichment of PIC with interacting protein pairs indicates that it detects protein-protein interactions rather than localization.

It should be noted that the gold standard negative set that we used for *S. cerevisiae *is made of protein pairs that do not co-localize. Therefore, it may be possible that PIC recognizes subcellular localization of proteins instead of protein-protein interactions. To examine this, we compiled a set of protein pairs that localize within the same subcellular compartment. Then, we assessed the enrichment of interacting protein pairs and co-localized protein pairs in the positive predictions of PIC at different thresholds. As Figure 2 shows, the PIC predictions are rapidly enriched by true interacting proteins rather than proteins that are localized in the same subcellular compartment. We also compiled an alternative standard negative set by using pairs of proteins that have Kyoto Encyclopedia of Genes and Genomes (KEGG) information [19], but do not share any KEGG pathway. Although this negative set is not as reliable as the main gold standard negative set that we used for the training and testing of PIC, it allows pairs of proteins that reside within the same subcellular compartment. The performance of PIC over this negative set was essentially the same as over the main gold standard negative set. For the other two studied organisms, *E. coli *and *P. falciparum*, the gold standard negative sets already contained co-localizing protein pairs.

Although PIC considers the relative frequencies of codons in ORF pairs, it reflects not only synonymous codon usage, but also amino acid frequencies and ORF lengths. ORF length is reflected in PIC since stop codons are not omitted, and each ORF has only one stop codon. Therefore, the relative frequency of a stop codon in long ORFs is smaller than in short ORFs. We created three other probabilistic interaction networks of *S. cerevisiae *using RSCU [20], relative frequencies of amino acids, and ORF length in order to examine the effect of each factor. We named these probabilistic networks PI-RSCU, PI-A and PI-L, respectively. RSCU is a measure of synonymous codon usage that is independent of amino acid composition (see reference [20] for the definition of RSCU and to compare it with the relative frequency of codon. RSCU as well as many other measures of synonymous codon usage are dependent on gene length, and result in biased values when the corresponding coding sequences are short [21]. In the worst case, when an amino acid is absent from a gene, it is impossible to calculate the RSCU for its corresponding codons. In the latter case, we treated the RSCU values of these codons as missing data, which can be easily handled by naïve Bayesian networks. In comparable sensitivities, the descending order of accuracy was PIC > PI-RSCU > PI-A > PI-L (Additional data file 5). This suggests a synergistic effect of each of these factors on the strength of PIC, with synonymous codon usage being the most important one. It should be mentioned that the length of the protein (PI-L) has a very marginal ability to distinguish interacting from non-interacting pairs, and even this observed marginal prediction may be due to the bias of the gold standard positive set towards a certain range of protein lengths, as the length of a protein affects many experimental procedures, such as successful cloning, and so on.

PIC can easily be combined with other probabilistic approaches, such as PIP (PI-predicted) and PIT (PI-total) [22] (see Materials and methods for combining two probabilistic interactomes). PIP is a probabilistic predicted network of *S. cerevisiae *in which four datasets of genomic features are integrated: two datasets of biological functions, a dataset of mRNA expression correlation and a dataset of essentiality [22]. Jansen *et al*. [22] showed that, at comparable levels of sensitivity, PIP is even more accurate than PIE (PI-experimental), a probabilistic network constructed by integration of four experimental datasets of the yeast interactome. They also combined PIP and PIE into PIT as one of the most comprehensive probabilistic networks of known and putative protein complexes in yeast. We integrated the results of yeast PIC and PIP to see how their combination improves our power in *de novo *prediction of interactions.

**Figure 3:**
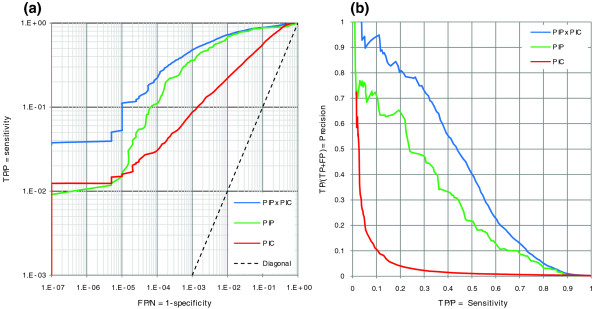
Comparison of performance in yeast for PIC, PIP and their combination. PIC is shown in red, PIP [22] in green and the combination of PIP and PIC (PIP × PIC) in blue. **(a) **Receiver operating characteristic (ROC) curves. Both axes are on log-scale. The dashed line shows the diagonal. **(b) **Precision-recall curves. FP, false positive; N, negative; P, positive; TP, true positive. Positive and negative test sets are as indicated in Table 1.

PIC, PIP [22] and their combination are compared in Figure 3. For false positive rates <10^-5^, PIC is as sensitive as PIP, although in general PIP is far superior to PIC. More strikingly, combining PIP and PIC results in a four-fold increase in sensitivity when the false positive rate is <10^-5 ^(after adding ribosomal proteins to the test set, a six-fold increase was observed). The combination of PIP and PIC remains the superior predictor for all false positive rates, and gets to a sensitivity of about 1.75 times that of PIP at a precision of 50%. Jansen *et al*. [22] used a likelihood threshold of 600 to cut an interaction network of *S. cerevisiae *out of PIP, referred to here as PIP-Lcut_600_. For comparable specificity, the combination of PIP and PIC is 1.5 times more sensitive than PIP-Lcut_600 _(considering ribosomal proteins in the test set, the combination of PIP and PIC is 1.6 times more sensitive than PIP-Lcut_600_; Additional data file 6). We also calculated the per-complex sensitivity of predictions for either PIP or the combination of PIP and PIC, and observed that the combination of PIP and PIC outperforms PIP in every single complex as well (Additional data file 7). Furthermore, we found that, compared to PIP, PIC in yeast is less biased towards certain biological functions (Additional data file 8) as well as highly expressed genes (Additional data file 9). However, it is evident that at least in the case of *P. falciparum *(Additional data file 14), PIC top-scoring interactions mainly belong to the ribosomal proteins. This reflects the very similar codon usage profiles of ribosomal proteins, most likely optimized for their efficient translation.

Finally, we combined PIT [22] and PIC to generate 'PICT', which we propose as one of the most reliable probabilistic interactomes of *S. cerevisiae *(see Additional data file 11 for precision-recall curves of PIT and PICT. PICT, accompanied by PIC for the whole genome of *S. cerevisiae*, is available online [23]). At a likelihood cutoff of 2 × 10^3^, PICT has the same specificity as PIT-Lcut_600_, while, after excluding promiscuous nodes (that is, nodes each of which has ≥100 edges), it includes 1,306 more ORFs compared to PIT. Analysis of PICT-Lcut_2000 _reveals many interesting interactions not present in PIT-Lcut_600_. Some examples are represented below. We specifically consider complexes that were also examined by Jansen *et al*. [22] in order to provide a more detailed comparison between PIT and PICT. Note that the following interactions should be considered as complex co-memberships rather than direct physical interactions, since all the components of PICT are trained on protein complexes and not one-to-one physical interactions of proteins. However, a direct physical interaction is also possible based on the closeness of proteins within the same complex.

**Figure 4:**
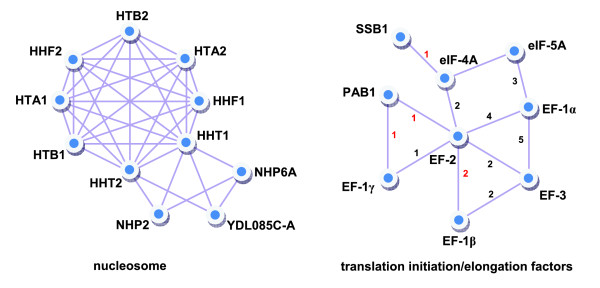
Two examples of complexes suggested by PICT-Lcut_2000_. In the case of translation initiation/elongation factors, only novel interactions (interactions absent from PIT-Lcut_600 _[22]) are represented. A black number between two nodes stands for the reference in which the direct interaction of the two connected nodes is reported. A red number refers to the reference in which interaction of the two connected nodes with a third common protein is reported. 1, Gavin *et al*. [27]; 2, Collins *et al*. [26]; 3, Jao and Chen [28]; 4, Jansen *et al*. [22]; 5, Anand *et al*. [29].

While mammalian Pob3, an interacting partner of the nucleosome, has a high mobility group (HMG) for interaction with histones, yeast Pob3 lacks this domain [22]. Instead, in yeast, the HMG protein Nhp6 interacts with the nucleosome. PIT-Lcut_600 _suggests that Nhp6A, an isoform of Nhp6, interacts with all nucleosome histones H2A, H2B, H3 and H4, which is highly unlikely considering the structure of the nucleosome. In addition, it has been shown that Nhp6 does not influence nucleosome reassembly; thus, it is unlikely for Nhp6 to interact with the H2A-H2B dimer [22]. In contrast to PIT-Lcut_600_, PICT-Lcut_2000 _only suggests an interaction between Nhp6A and HHT1 (H3), which is more congruent with the current models of nucleosome structure and assembly. PICT-Lcut_2000 _also predicts a novel interaction between Nhp2, another HMG related protein, and H3 (Figure 4). Recently, affinity capture of Nhp2 has been shown to result in co-purification of histone proteins [24], corroborating the interaction of this protein with the nucleosome. PICT-Lcut_2000 _also predicts the interaction of an uncharacterized ORF, YDL085C-A, with the nucleosome as well as with Nhp6A, which is consistent with previous reports showing the presence of GFP-fused YDL085C-A in the nucleus [25]. This example shows the potential of PICT, and codon usage in particular, to predict interactions of uncharacterized proteins, which should provide new insights into their probable functions.

Another example is the case of translation initiation/elongation factors. PIT-Lcut_600 _fails to predict an interaction involving elongation factor 2 (EF-2). It also predicts only two interactions for EF-1α, with EF-1β and EF-1γ. Although PIT-Lcut_300 _suggests some more interactions for these proteins, a higher rate of false positives in PIT-Lcut_300 _renders them unreliable. PICT-Lcut_2000 _predicts several interactions involving different elongation factors as well as initiation factors 4A and 5A, many of which have been recently confirmed by tandem-affinity purification experiments [22,26-29]]. Figure 4 shows a subgraph of PICT-Lcut_2000 _representing interactions among translation initiation/elongation factors that are not present in PIT-Lcut_600_. A recent study [27] has shown that Poly(A)-binding protein Pab1 interacts with EF-1α. Based on PICT-Lcut_2000_, we anticipate that Pab1 interacts with EF-2 and EF-1γ as well. Also, we found an interesting interaction between the ribosome-associated molecular chaperone Ssb1 and eIF4A. Interaction of Ssb1 and eIF4G has already been shown by tandem-affinity purification [27]. Based on the close interaction of eIF4A and eIF4G, interaction of Ssb1 and eIF4A is reasonable.

RNase P complex represents another interesting example of PICT predictions. PICT-Lcut_2000 _predicts six new interactions between RNase P complex and other proteins in yeast, neither of which exists in PIT-Lcut_600 _or has been reported previously. Four interactions are with uncharacterized ORFs, YKL096C-B, YDL159W-A, YKL183C-A and Q0255. Q0255 is likely to code for a maturase-like protein. It has been hypothesized that mitochondrial maturases participate in splicing by stabilizing some secondary or tertiary structure needed for splicing [30]. Their exact function, however, remains uncharacterized [31]. An interaction between RNase P complex and Q0255 implies the plausibility that this protein could contribute to maturation of ribosomal RNA and tRNA in mitochondria. According to PICT-Lcut_2000_, HUB1 (Histone mono-ubiquitination 1) is another interacting partner of RNase P complex. Previous data have shown that HUB1 is a functional homolog of the human and yeast BRE1 proteins, and suggest that it mediates gene activation and cell cycle regulation through chromatin modifications [32]. In addition, chromatin remodeling in *Arabidopsis thaliana *seed dormancy is proposed to be mediated by H2B mono-ubiquitination through HUB1 and HUB2 [32]. In agreement with this, the recently reported binding of human RNase P to chromatin of non-coding RNA genes and regulation of pol III transcription [33] could be mediated through a HUB1-RNase P interaction. Another prediction of PICT-Lcut_2000_, interaction of RNase P with CKB1, also corroborates this observation. CKB1 is a regulatory subunit of casein kinase 2, whose many substrates include transcription factors and all RNA polymerases. Again, this is consistent with the recent proposed role for RNase P in pol III transcription [33,34].

We notice that PICT has the potential of providing new information about proteins that lack homology. For example, YAR068W is a fungal-specific gene, for which PIT has no interaction. This is while PICT predicts an interaction between this protein and a protein of the large subunit of mitochondrial ribosome (refer to PICT-Lcut_2000 _in Additional data file 13).

## Conclusion

PIC uses a naïve Bayesian network to combine the information provided by the frequencies of all codons in order to predict protein-protein interactions. Given a set of independent features, naïve Bayesian networks can combine them in a way that minimizes the loss of information that usually occurs by the aggregation of several features. Depending on the training set that has been used, PIC can predict both complex membership (as in the Munich Information Center for Protein Sequences (MIPS) database or TAP-tagging experiments) and functional linkages between proteins (as in the KEGG pathway database). Although we did not test the power of PIC for prediction of direct physical interactions between proteins, it is possible that it can be used for that purpose as well, since complex membership, functional linkage and direct physical interactions are all properties that are highly inter-correlated. We anticipate that integrating PIC with the current knowledge of protein interactions in different organisms will significantly increase the reliability and coverage of probabilistic interactomes. In the case of *S. cerevisiae*, the results of PIC as well as its combination with PIT [22], referred to in this article as PICT, are provided online [23]. This study not only describes a novel method for *de novo *prediction of protein-protein interactions, but also suggests the plausibility of previously unseen evolutionary forces acting on codon compositions of genes within a genome. A few studies have taken into account the effect of protein-protein interactions on codon usage; however, these studies generally consider the unique features of codon composition of an ORF in regions that code the interacting face of the protein compared to the rest of the ORF [35], not the direct relationship between codon usages of two interacting proteins. Characterization of evolutionary mechanisms shaping these relationships may lead to development of even more powerful methods for sequence-based prediction of interaction networks.

## Materials and methods

### Genome sequences

The genome sequences used were *S. cerevisiae *[36], *E. coli *[37] and *P. falciparum *[38].

### Analysis of genomic features

We used *d*_*ij*_(*ζ*^*k*^) = |*ζ*^*k*^_*i *_- *ζ*^*k*^_*j*_| to measure the distance of two genes *i *and *j *regarding feature *ζ*^*k*^. In the case of PIC, *ζ*^*k *^= *f*(*c*_*k*_), where *f*(*c*_*k*_) is the normalized frequency of usage of codon *c*_*k*_, so that Σ_*k*_*f *(*c*_*k*_) = 1 (1 ≤ *k *≤ 64). For PI-RSCU, *ζ*^*k *^= RSCU(*c*_*k*_) (see [20]). For PI-A, *ζ*^*k *^= *f*(*a*_*k*_), where *f*(*a*_*k*_) is the normalized frequency of amino acid *a*_*k *_(1 ≤ *k *≤ 20). For PI-L, *ζ *= L, where L represents the ORF length. To combine a set of features, a naïve Bayesian network [13] is employed. Naïve Bayesian networks are most effective when they are used to combine independent features. We assessed independency of *d*_*ij *_for two features *r *and *s *by means of mutual information [13], where *I *[*d*_*ij*_(*ζ*^*r*^);*d*_*ij*_(*ζ*^*s*^)] < 0.01 was assumed not to influence the performance of the naïve Bayesian network. To combine two probabilistic networks, we multiplied the likelihoods each network assigned to each interaction.

### Coevolution of CAI

We performed the same analysis as described by Fraser *et al*. [14], using the genome sequences of *S. cerevisiae*, *Saccharomyces paradoxus*, *Saccharomyces mikatae*, and *Saccharomyces bayanus *[39]. We used species-specific adaptation index to determine the CAI values by using the codon frequencies of the 20 most highly expressed genes. We assumed that the 20 most highly expressed genes in the four species are the same; hence, we used a previous report on mRNA expression in *S. cerevisiae *[40] to identify them. Addition of *E. coli *in the analysis did not improve the results. We did not add more genomes because we would lose a portion of our gold standard sets, especially the negative gold standard set, due to the lack of homology for all genes among all genomes, resulting in non-comparable sensitivity/specificity values.

## Abbreviations

CAI, codon adaptation index; EF, elongation factor; HMG, high mobility group; HUB, Histone mono-ubiquitination; KEGG, Kyoto Encyclopedia of Genes and Genomes; Lcut, likelihood cutoff; MIPS, Munich Information Center for Protein Sequences; ORF, open reading frame; PI, probabilistic interactome; PI-A, PI using amino acid frequencies; PIC, probabilistic-interactome using codon usage; PICT, combination of PIC and PIT; PIE, PI-experimental; PI-L, PI using sequence length; PIP, PI-predicted; PI-RSCU, PI using RSCU; PIT: PI-total.

## Authors' contributions

HSN and RS contributed to all aspects of this research. Both authors read and approved the final manuscript.

## Additional data files

The following additional data are available with the online version of this paper. Additional data file 1 is a figure showing the distribution of *d *for each codon in yeast. Additional data file 2 is a figure comparing the naïve Bayesian network and fully connected Bayesian network in the yeast gold standard positive set. Additional data file 3 is a figure comparing the naïve Bayesian network and fully connected Bayesian network in the yeast gold standard negative set. Additional data file 4 demonstrates the variance over different components resulting from principal component analysis of the interacting gene pairs in yeast. Additional data file 5 compares PIC, PI-RSCU, PI-A and PI-L in a figure. Additional data file 6 is a figure comparing PIP × PIC and the yeast gold standard positive set. Additional data file 7 illustrates per-complex comparison of PIP and PIP × PIC in a figure. Additional data file 8 is a figure showing the MIPS functional category enrichment for the yeast genome, PIP-Lcut_600 _and PIC-Lcut_600_. Additional data file 9 is a figure representing the distribution of mRNA expression levels in interactions predicted by PIP-Lcut_600 _and PIC-Lcut_600 _for *S. cerevisiae*. Additional data file 10 shows the precision-recall curves for PIC, interolog mapping (INT), phylogenetic profiles (PGP), Rosetta stone (ROS), CAI coevolution (co-CAI) and CAI. Additional data file 11 includes precision-recall curves for PIC, PIT and PICT. Additional data file 12 is a compressed file containing PIC-Lcut_600 _for *S. cerevisiae*. Additional data file 13 is a compressed file containing PICT-Lcut_2000 _for *S. cerevisiae*. Additional data file 14 is a compressed file containing the results of performance of PIC on the *P. falciparum *gold standard set.

## Supplementary Material

Additional data file 1 Distribution of *d *for each codon in yeast. Click here for file

Additional data file 2 Comparison of the naïve Bayesian network and fully connected Bayesian network in the yeast gold standard positive set. Click here for file

Additional data file 3 Comparison of the naïve Bayesian network and fully connected Bayesian network in the yeast gold standard negative set. Click here for file

Additional data file 4 Variance over different components resulting from principal component analysis of the interacting gene pairs in yeast. Click here for file

Additional data file 5 Comparison of PIC, PI-RSCU, PI-A and PI-L. Click here for file

Additional data file 6 Comparison of PIP × PIC and the yeast gold standard positive set. Click here for file

Additional data file 7 Per-complex comparison of PIP and PIP × PIC. Click here for file

Additional data file 8 MIPS functional category enrichment for the yeast genome, PIP-Lcut_600 _and PIC-Lcut_600_. Click here for file

Additional data file 9 Distribution of mRNA expression levels in interactions predicted by PIP-Lcut_600 _and PIC-Lcut_600 _for *S. cerevisiae*. Click here for file

Additional data file 10 precision-recall curves for PIC, interolog mapping (INT), phylogenetic profiles (PGP), Rosetta stone (ROS), CAI coevolution (co-CAI) and CAI. Click here for file

Additional data file 11 Precision-recall curves for PIC, PIT and PICT. Click here for file

Additional data file 12 PIC-Lcut_600 _for *S. cerevisiae*. Click here for file

Additional data file 13 PICT-Lcut_2000 _for *S. cerevisiae*. Click here for file

Additional data file 14 Results of performance of PIC on the *P. falciparum *gold standard set. For the performance of PIC on *Escherichia coli *gold standard set check reference [23]. Click here for file
