# Supplementary material for: Sequence-based prediction of protein-protein interactions by means of codon usage
Source: Genome Biol. 2008 May 23;9(5):R87. doi: 10.1186/gb-2008-9-5-r87 (PMC2441473; doi:10.1186/gb-2008-9-5-r87)
Supplement: Additional data file 1 — Distribution of d for each codon in yeast. [file gb-2008-9-5-r87-S1.pdf]

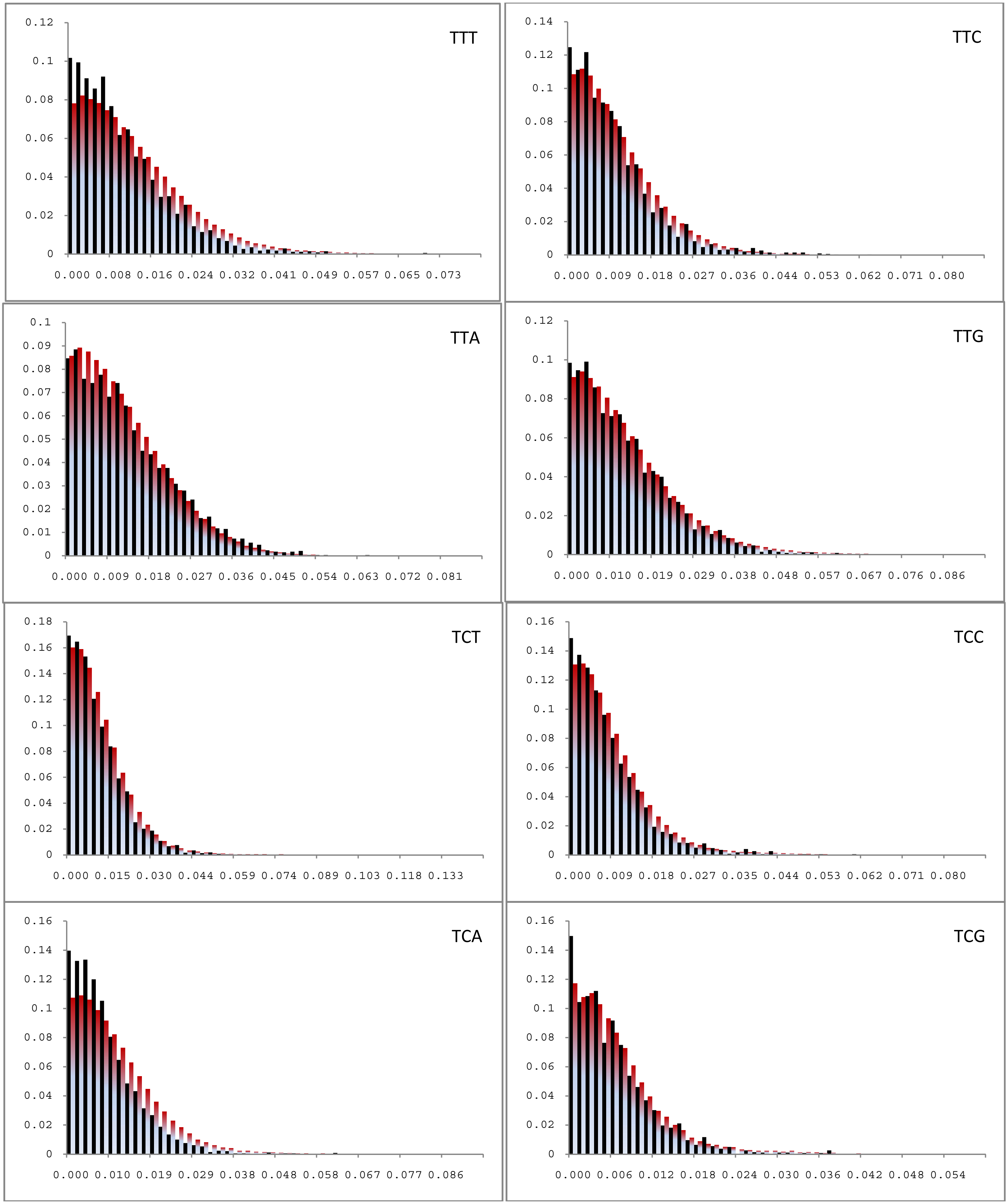

Figure S1 Distribution of *d* for each codon in yeast. The value of *d* is demonstrated on the horizontal axis, whereas the vertical axis shows the density. Black bars represent distribution of *d* in positive gold standard set and red bars stand for the distribution of *d* in negative gold standard set.

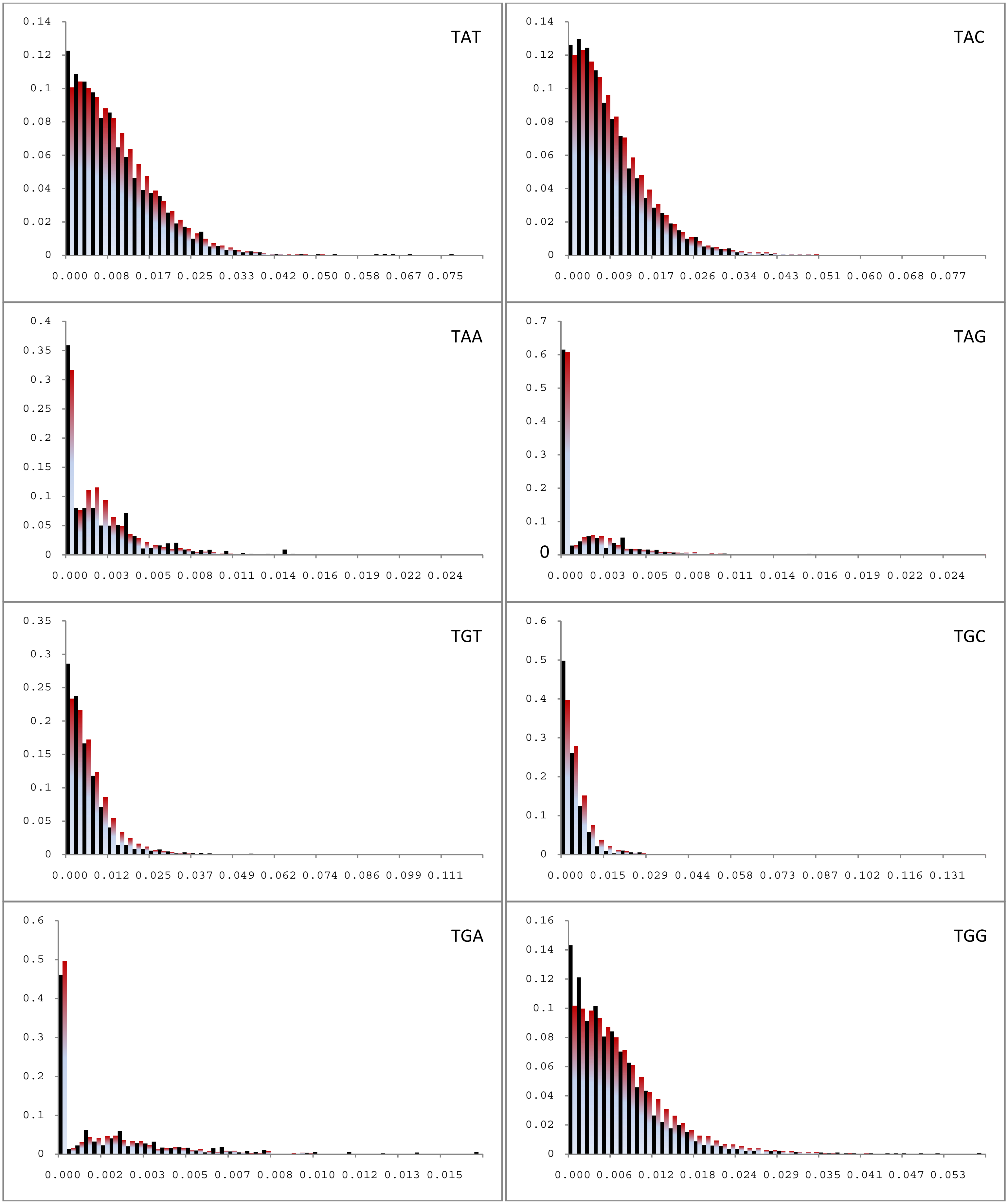

Figure S1 Continued.

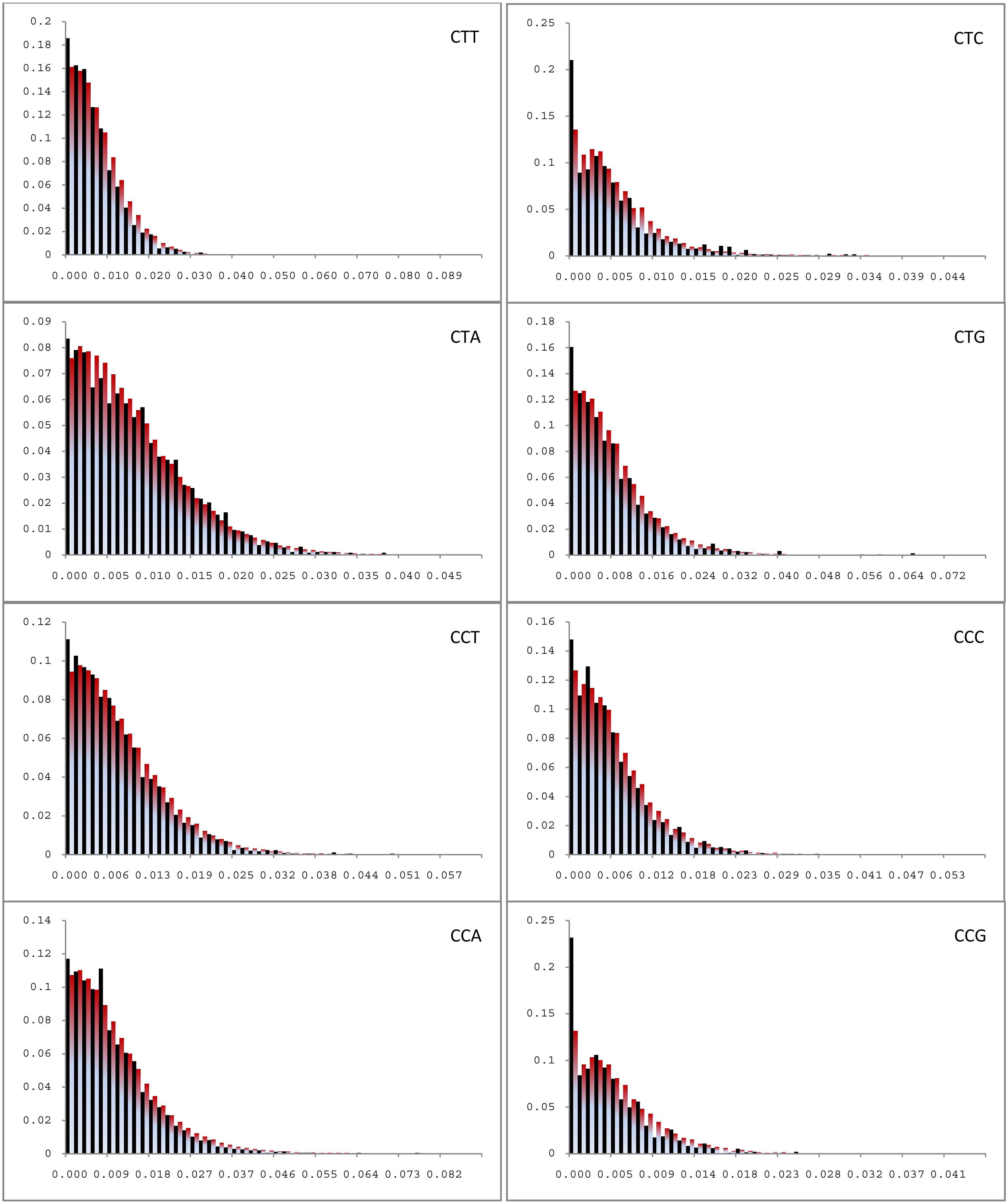

Figure S1 Continued

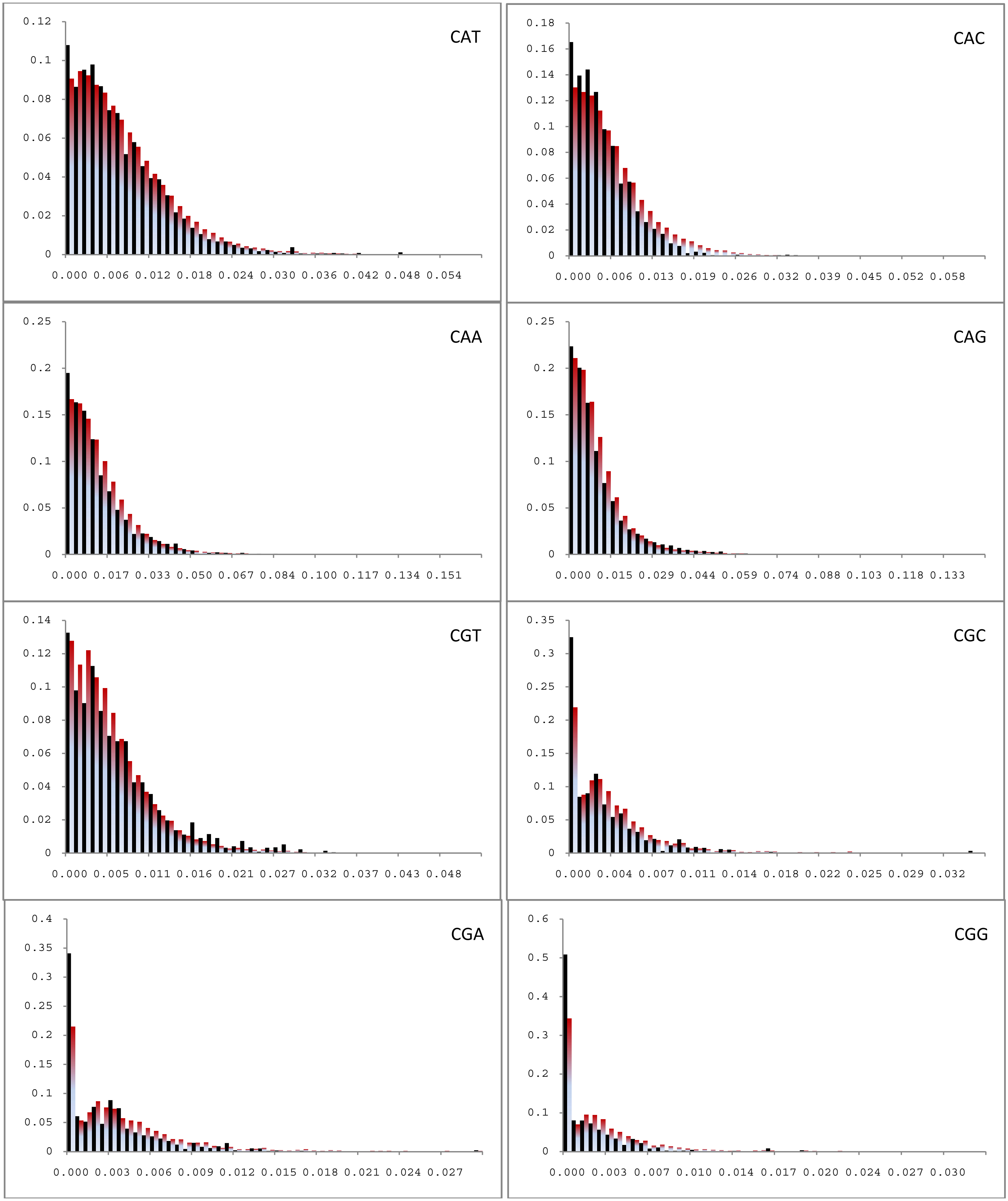

Figure S1 Continued

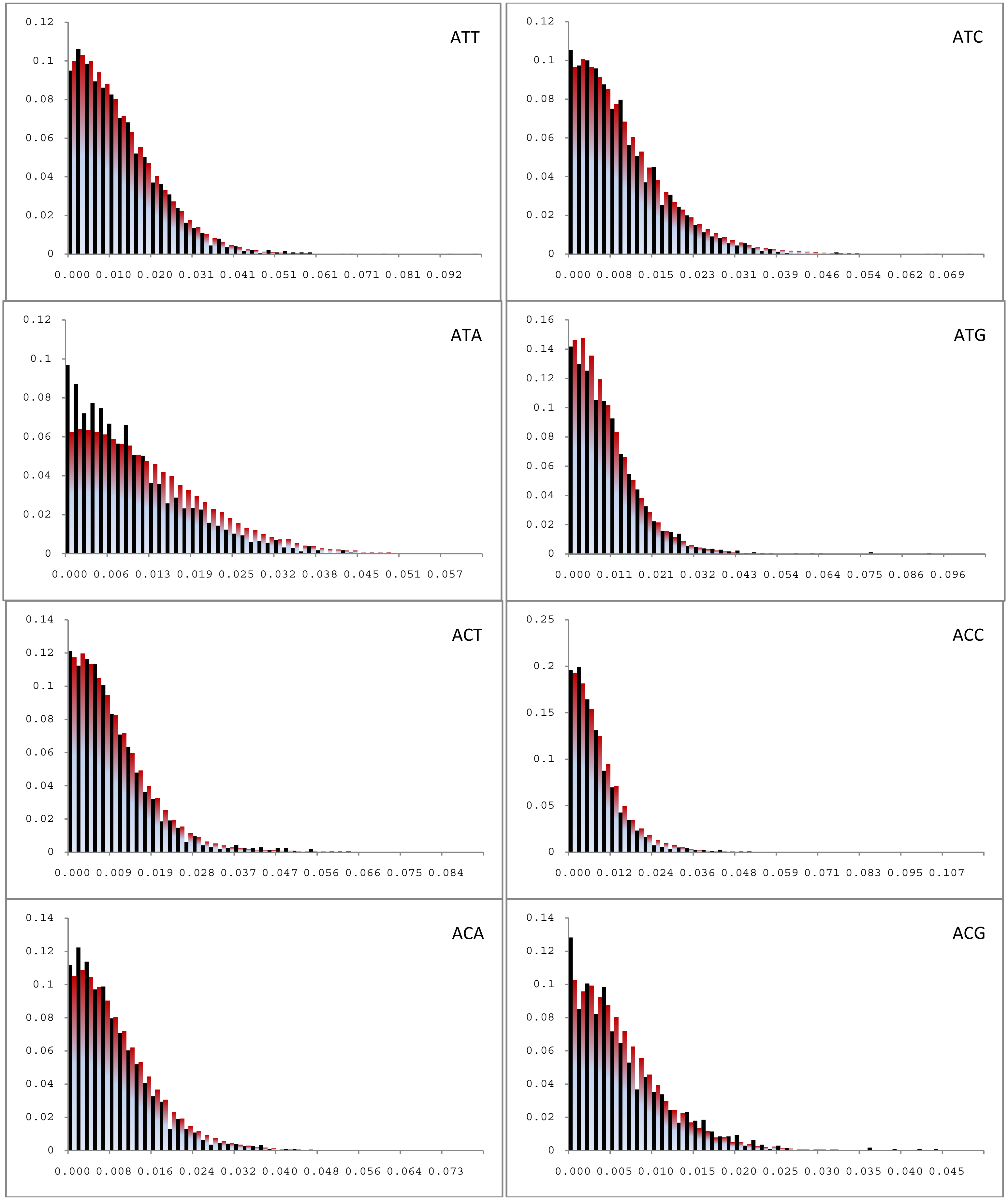

Figure S1 Continued

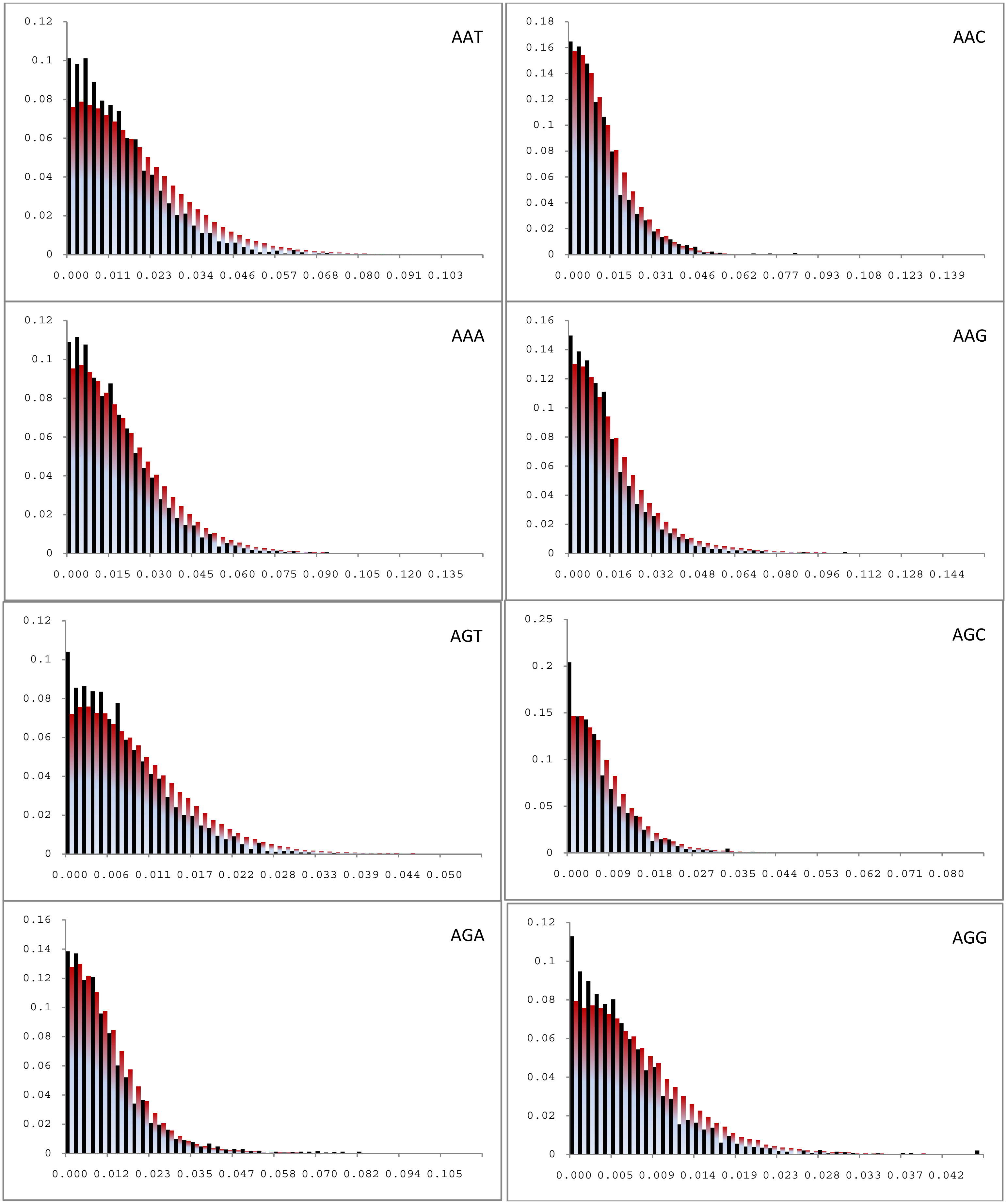

Figure S1 Continued

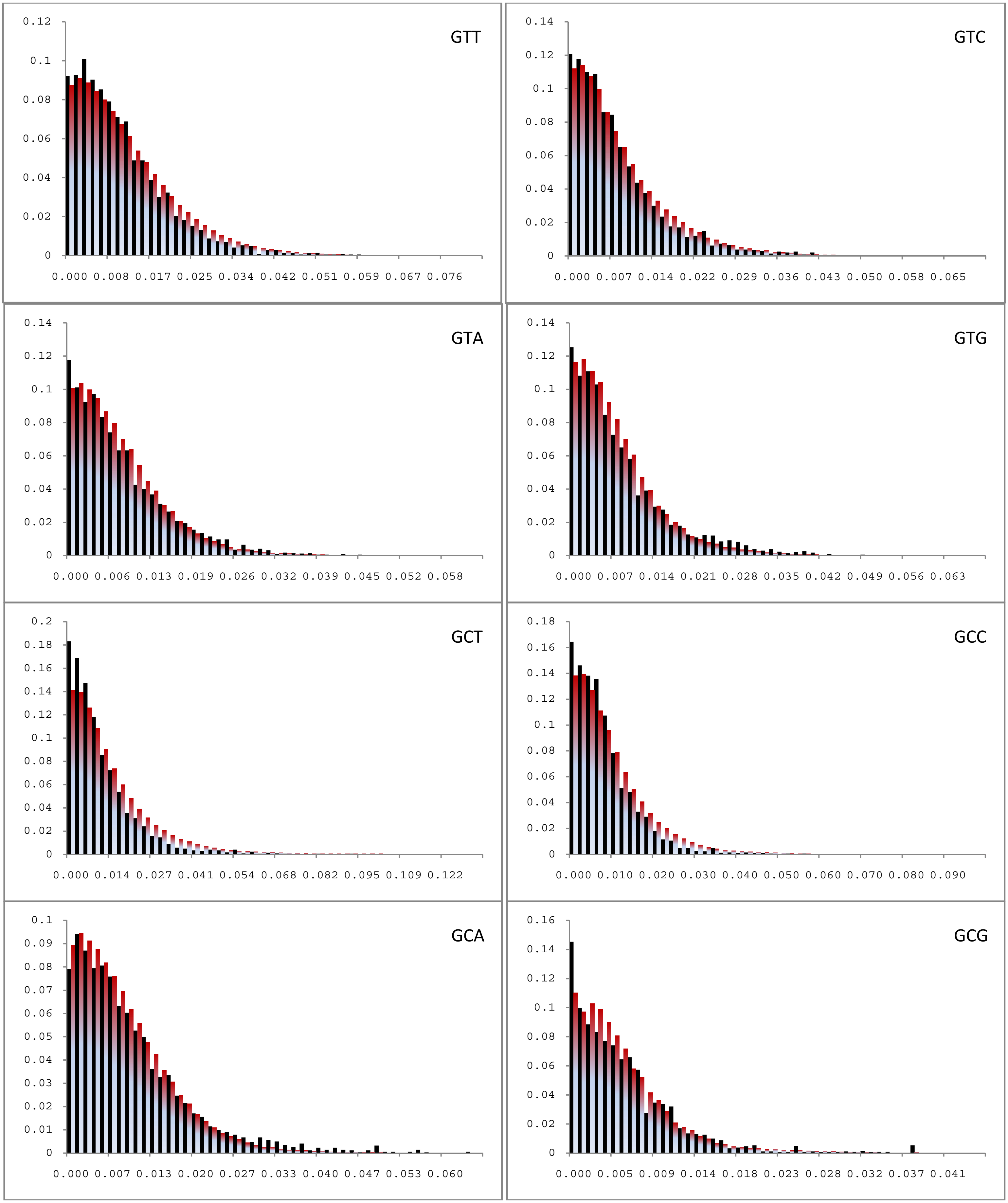

Figure S1 Continued

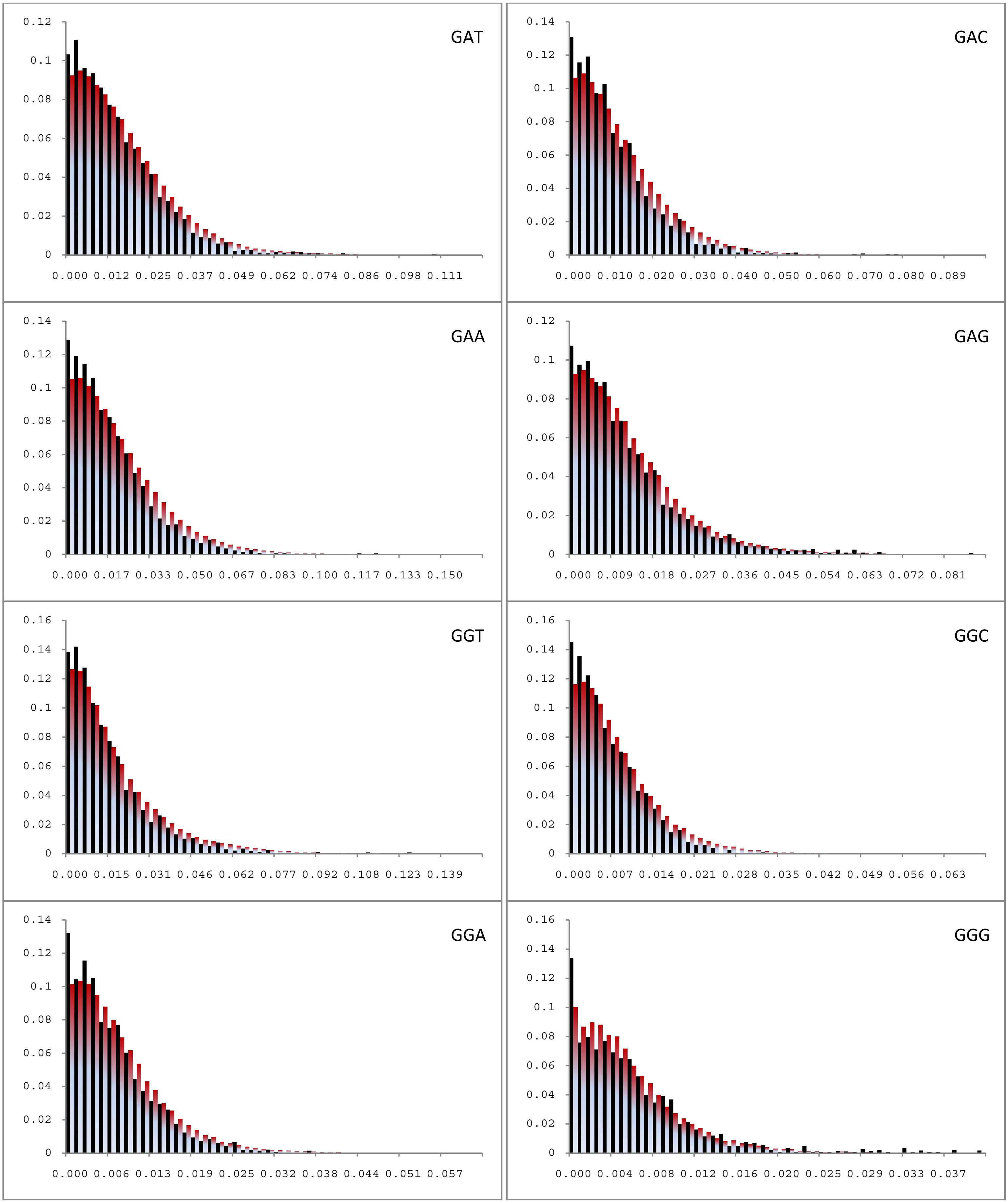

Figure S1 Continued
