# Supplementary material for: Sequence-based prediction of protein-protein interactions by means of codon usage
Source: Genome Biol. 2008 May 23;9(5):R87. doi: 10.1186/gb-2008-9-5-r87 (PMC2441473; doi:10.1186/gb-2008-9-5-r87)
Supplement: Additional data file 3 — Comparison of the naïve Bayesian network and fully connected Bayesian network in the yeast gold standard negative set. [file gb-2008-9-5-r87-S3.pdf]

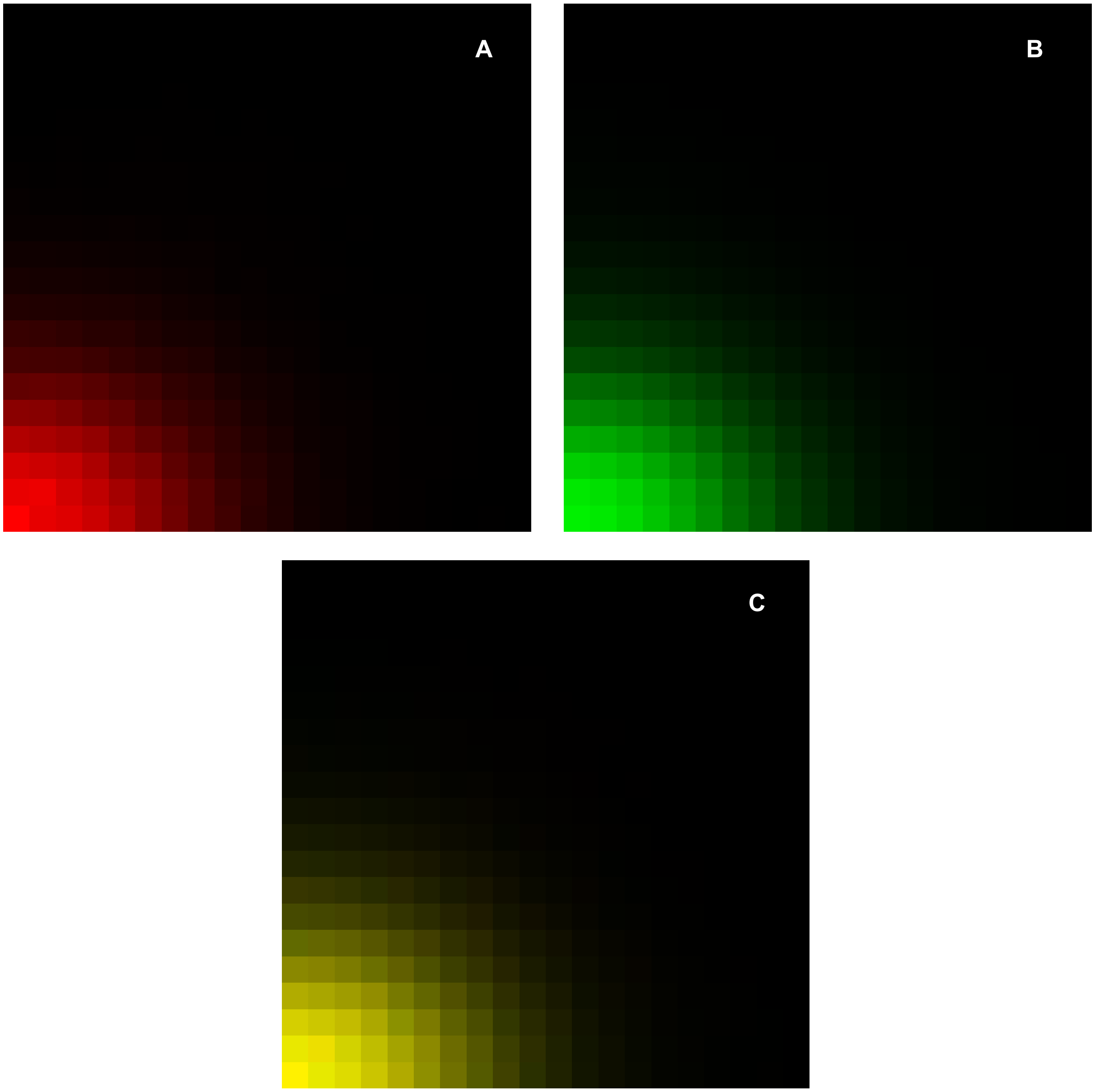

Figure S3 Comparison of naïve Bayesian network and fully connected Bayesian network in yeast gold standard negative set. In each panel, the horizontal axis shows *d*(TTT) and the vertical axis shows *d*(TTC). Color intensity represents the probability of a non-interacting pair of proteins having the respective *d*(TTT) and *d*(TTC) values, predicted by either fully connected Bayesian network (shown in red, panel **A**) or naïve Bayesian network (shown in green, panel **B**). Panel **C** shows the combination of panels **A** and **B** (yellow). The Naïve Bayesian network results in approximately the same distribution as the fully connected Bayesian network. The results are the same for almost all pairs of codons.
