# Supplementary material for: Sequence-based prediction of protein-protein interactions by means of codon usage
Source: Genome Biol. 2008 May 23;9(5):R87. doi: 10.1186/gb-2008-9-5-r87 (PMC2441473; doi:10.1186/gb-2008-9-5-r87)
Supplement: Additional data file 4 — Variance over different components resulting from principal component analysis of the interacting gene pairs in yeast. [file gb-2008-9-5-r87-S4.pdf]

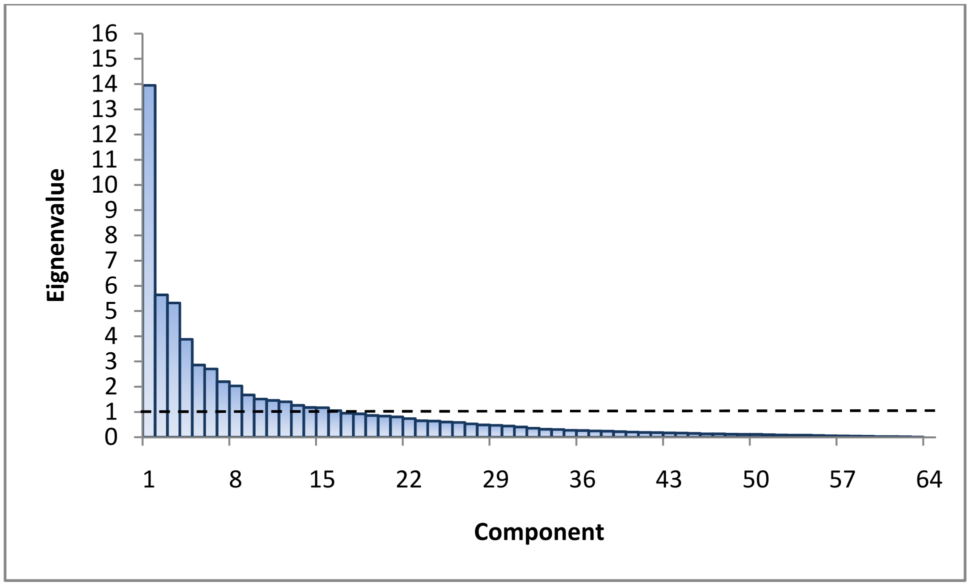

Figure S4 Eigenvalues of different components resulted from principal component analysis of the interacting gene pairs in yeast. A 64-dimensional space was constructed consisting of all interacting pairs of genes from yeast gold standard set, so that dimension *k* represents $d(c_k)$ for each pair of genes. Then, this space was transformed using principal component analysis (PCA). In this figure, the components are in decremental order of their eigenvalue, the first component being the principal component of the transformed space. The first 16 components have eigenvalues greater than unity, suggesting the presence of 16 effective components based on the Guttman-Kaiser criterion (Guttman, L., 1954. *Psychometrika* XIX:149-61). However, the Scree test (Cattell, R.B., 1966. *Multivariate Behavioural Research* 1:245-76) suggests the presence of about 10 effective components. We also calculated an entropy-based number of effective codons for the collection of all coding sequences in *S. cerevisiae*, formulated as $N = e^{-\sum_{k=1}^{64} f(c_k) ln[f(c_k)]}$, yielding in a value of 52.23. Both the effective number of components obtained from the Guttman-Kaiser criterion and the Scree test are small compared to the entropy-based number of effective codons, suggesting that the differences of codon frequencies in yeast contain redundant information.
