# Supplementary figures and images for: Sequence-based prediction of protein-protein interactions by means of codon usage
Source: Genome Biol. 2008 May 23;9(5):R87. doi: 10.1186/gb-2008-9-5-r87 (PMC2441473; doi:10.1186/gb-2008-9-5-r87)

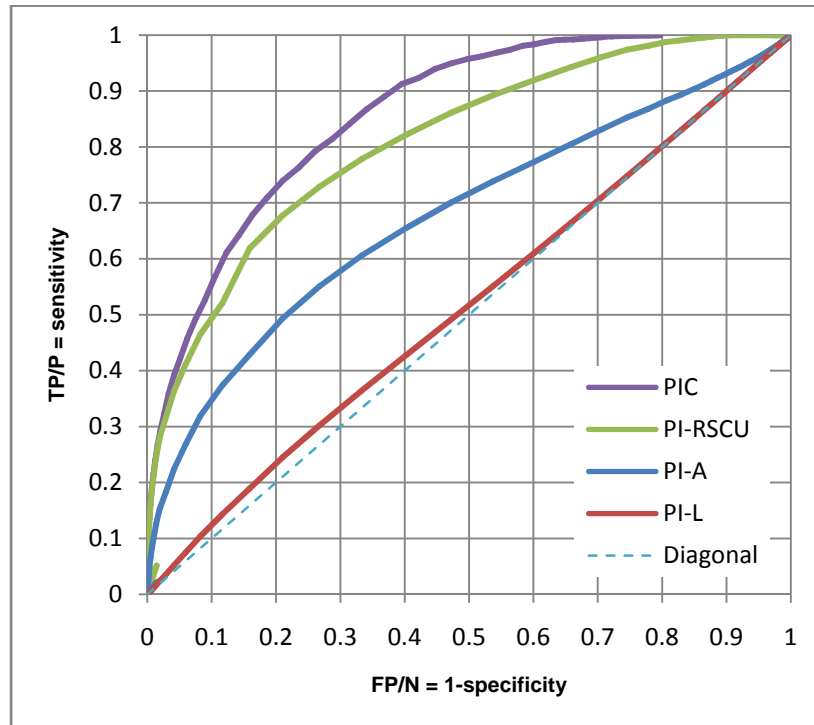

**Figure S5** Comparison of ROC curves for PIC, PI-RSCU, PI-A and PI-L.

Supplement: Additional data file 5 — Comparison of PIC, PI-RSCU, PI-A and PI-L. [file gb-2008-9-5-r87-S5.pdf]
