# Supplementary material for: Sequence-based prediction of protein-protein interactions by means of codon usage
Source: Genome Biol. 2008 May 23;9(5):R87. doi: 10.1186/gb-2008-9-5-r87 (PMC2441473; doi:10.1186/gb-2008-9-5-r87)
Supplement: Additional data file 6 — Comparison of PIP × PIC and the yeast gold standard positive set. [file gb-2008-9-5-r87-S6.pdf]

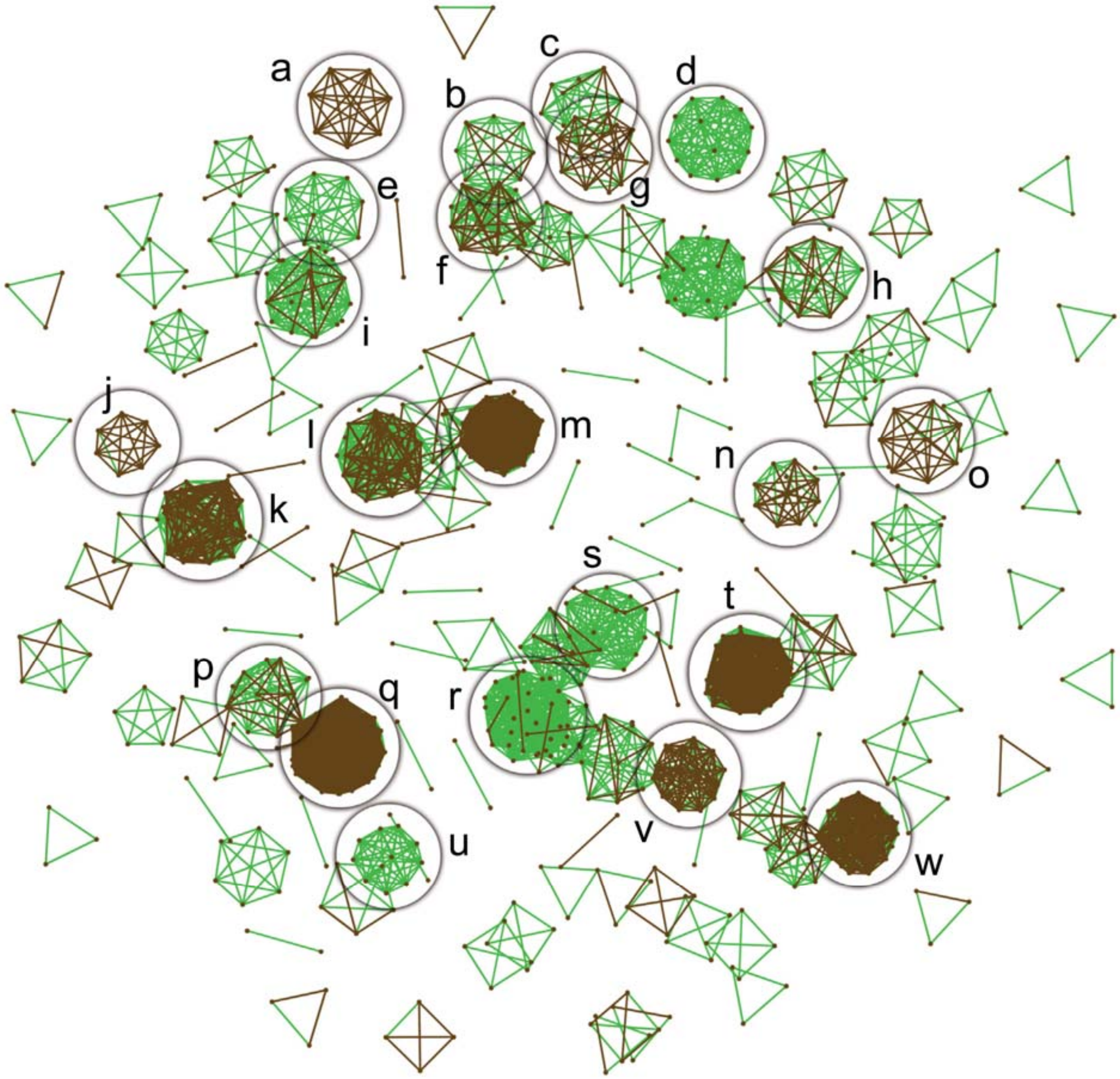

Figure S6. Comparison of PIP×PIC and yeast gold standard positive set (including ribosomal proteins). PIP×PIC (brown) covers 43% of the gold standard positives (green), while PIP has a coverage of 27% at the same specificity. (a) nucleosome; (b) COPI; (c) RNase MRP; (d) CCR4-NOT; (e) ER oligosaccharyl-transferase; (f) V-ATPase; (g) Cct-ring; (h) TFIIH; (i) small subunit of mitochondrial ribosome; (j) exocyst complex; (k) transcription; (l) pre-replication; (m) 40S ribosomal subunit; (n) ubiquinol cytochrome-c reductase; (o) cytochrome-c oxidase; (p) APC/C; (q) 60S ribosomal subunit; (r) RNA-polymerase II mediator; (s) SAGA complex; (t) proteasome; (u) TRAPP; (v) F1F0 ATP synthase; (w) large subunit of mitochondrial ribosome.
