# Supplementary material for: Sequence-based prediction of protein-protein interactions by means of codon usage
Source: Genome Biol. 2008 May 23;9(5):R87. doi: 10.1186/gb-2008-9-5-r87 (PMC2441473; doi:10.1186/gb-2008-9-5-r87)
Supplement: Additional data file 7 — Per-complex comparison of PIP and PIP × PIC. [file gb-2008-9-5-r87-S7.pdf]

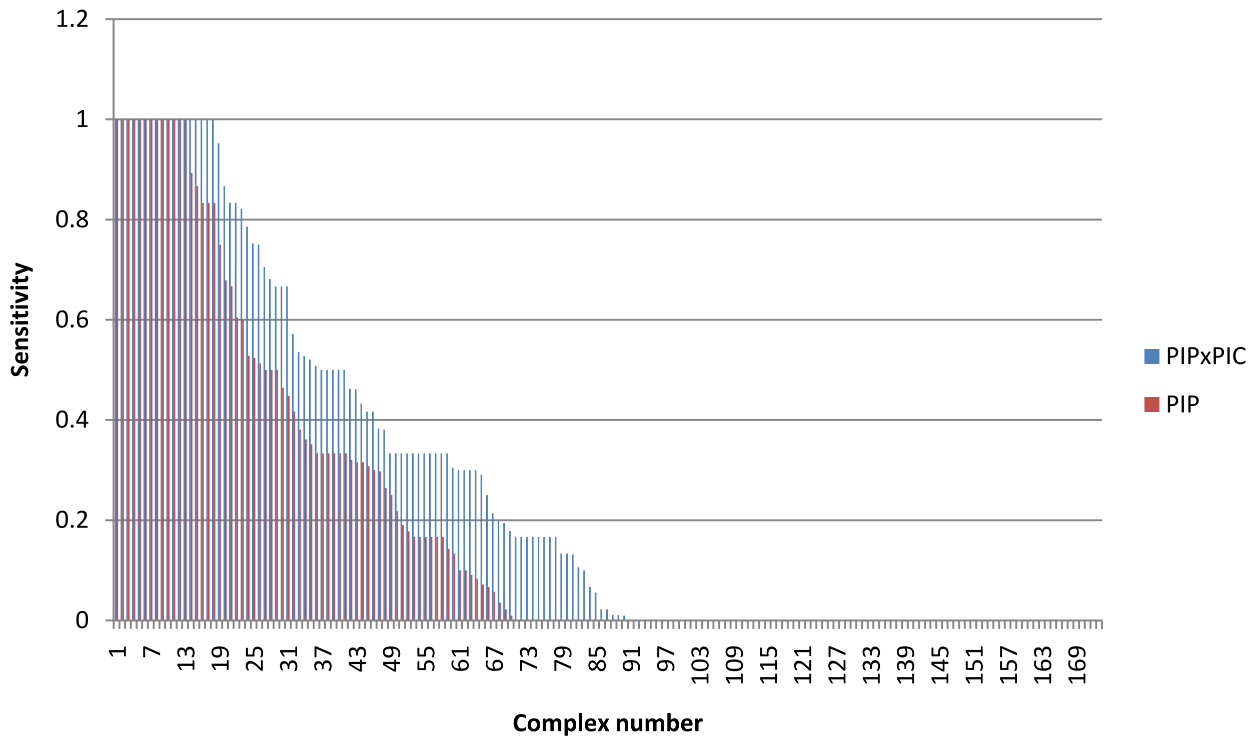

Figure S7 Per-complex comparison of PIP and PIPxPIC. The sensitivity of each of these two methods is given for each complex. The complex numbers are in the descending order of the sensitivity of PIP.
