# Supplementary material for: Sequence-based prediction of protein-protein interactions by means of codon usage
Source: Genome Biol. 2008 May 23;9(5):R87. doi: 10.1186/gb-2008-9-5-r87 (PMC2441473; doi:10.1186/gb-2008-9-5-r87)
Supplement: Additional data file 8 — MIPS functional category enrichment for the yeast genome, PIP-Lcut600 and PIC-Lcut600. [file gb-2008-9-5-r87-S8.pdf]

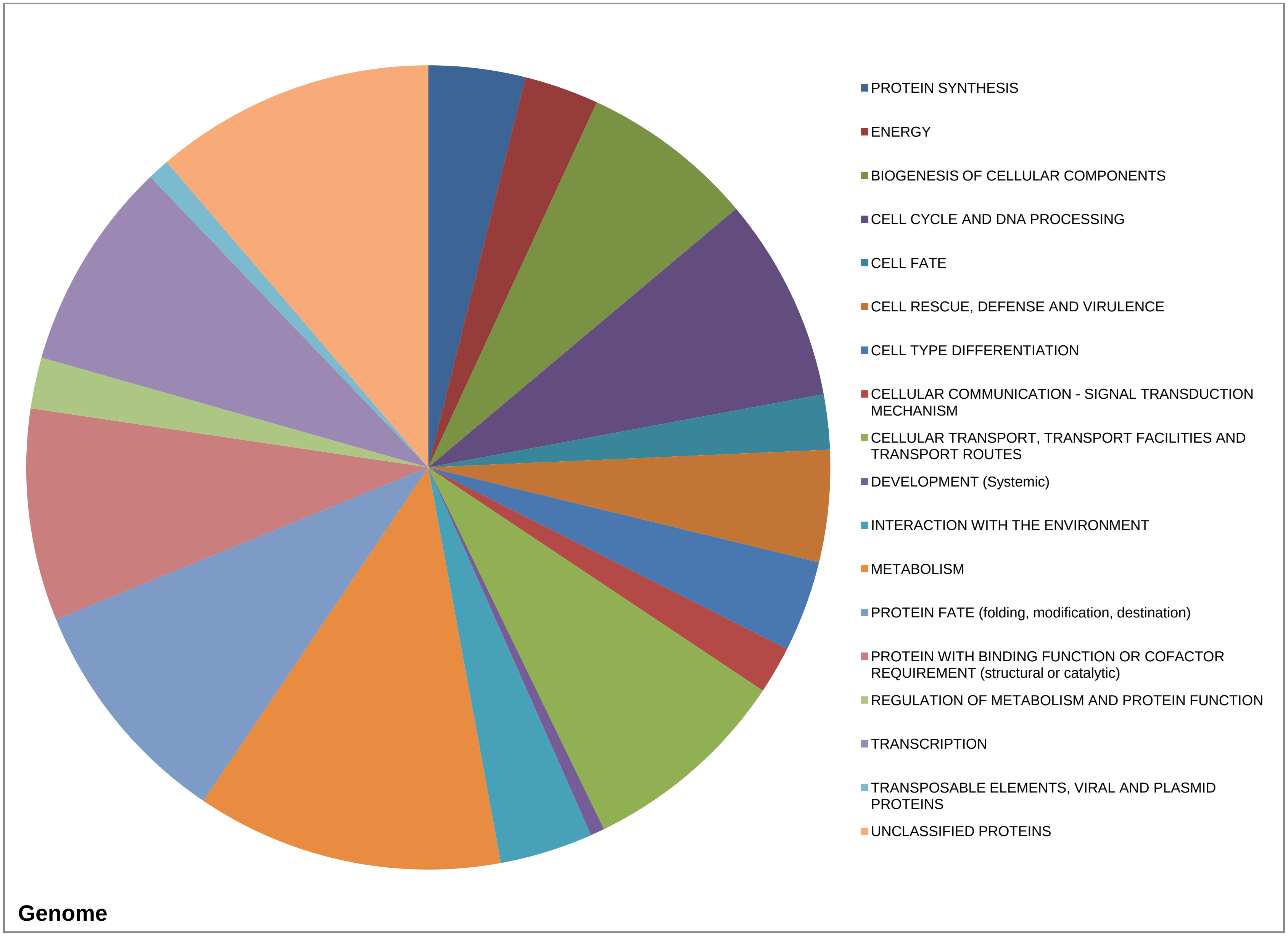

Figure S8 MIPS functional category enrichment for yeast genome, PIP-Lcut$_{600}$ and PIC-Lcut$_{600}$. Each slice reflects either the number of ORFs classified under the respective functional category (in the case of yeast genome), or the number of interactions involving at least one ORF from the respective category (in the case of PIP-Lcut$_{600}$ and PIC-Lcut$_{600}$). Both PIP-Lcut$_{600}$ and PIC-Lcut$_{600}$ are, compared to yeast genome, enriched by proteins employed for protein synthesis, even though the training set of PIC was deprived from ribosomal proteins. PIC-Lcut$_{600}$ contains many interactions involving proteins of unclassified function, while PIP-Lcut$_{600}$ includes few interactions of this kind, mainly because of its dependence on biological function as a predictor.

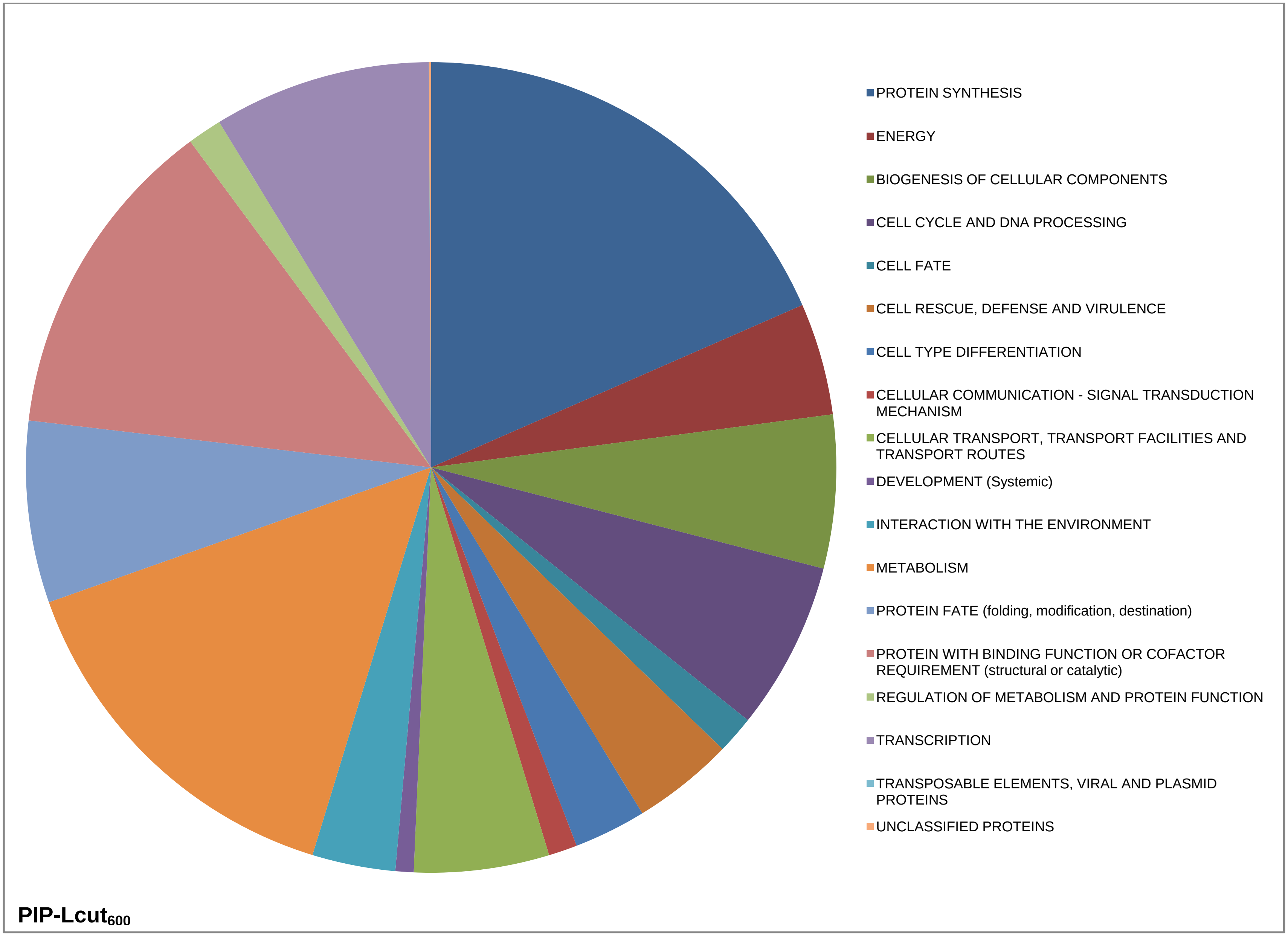

Figure S8 Continued

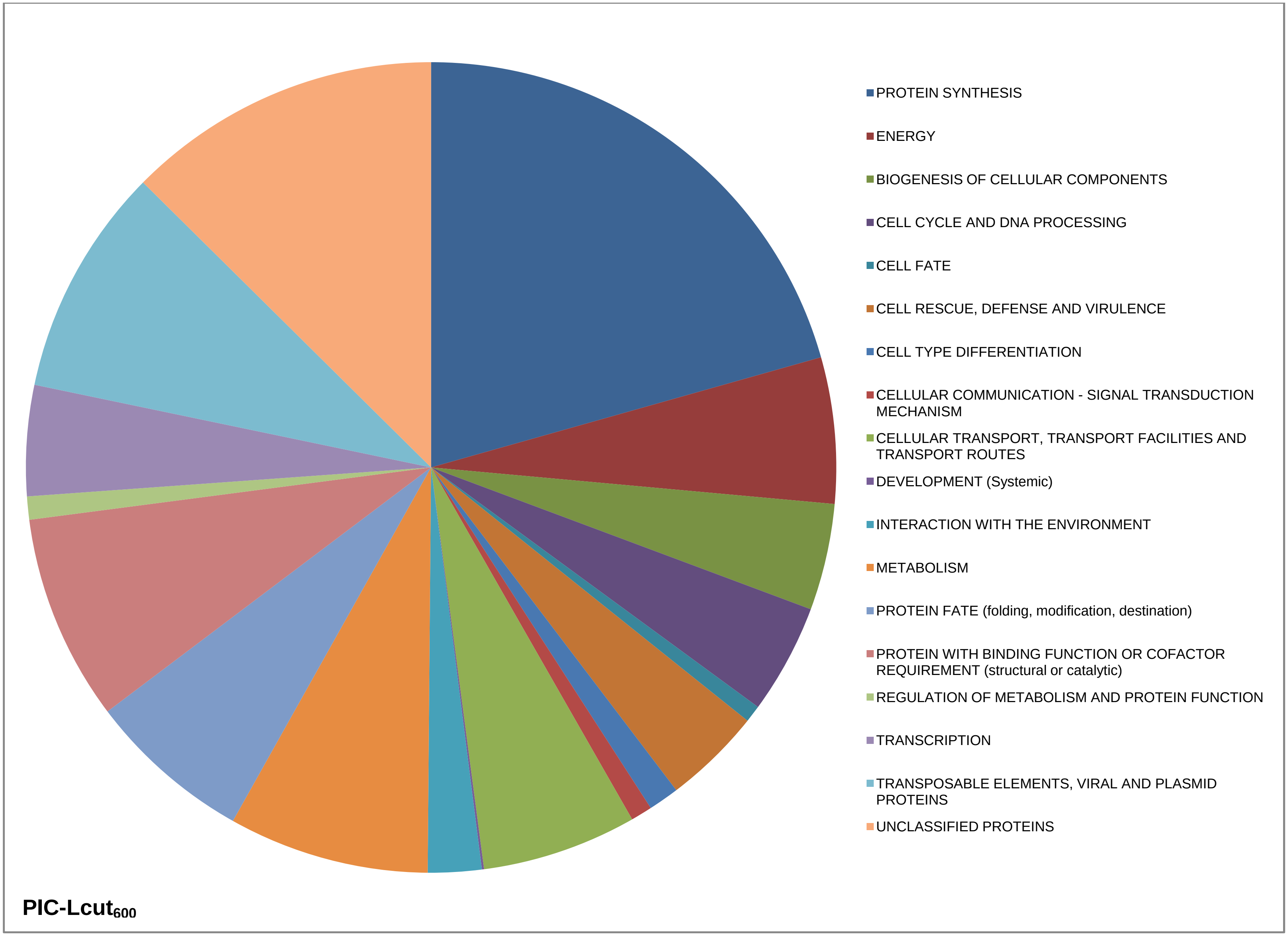

Figure S8 Continued
