# Supplementary material for: Sequence-based prediction of protein-protein interactions by means of codon usage
Source: Genome Biol. 2008 May 23;9(5):R87. doi: 10.1186/gb-2008-9-5-r87 (PMC2441473; doi:10.1186/gb-2008-9-5-r87)
Supplement: Additional data file 9 — Distribution of mRNA expression levels in interactions predicted by PIP-Lcut600 and PIC-Lcut600 for S. cerevisiae. [file gb-2008-9-5-r87-S9.pdf]

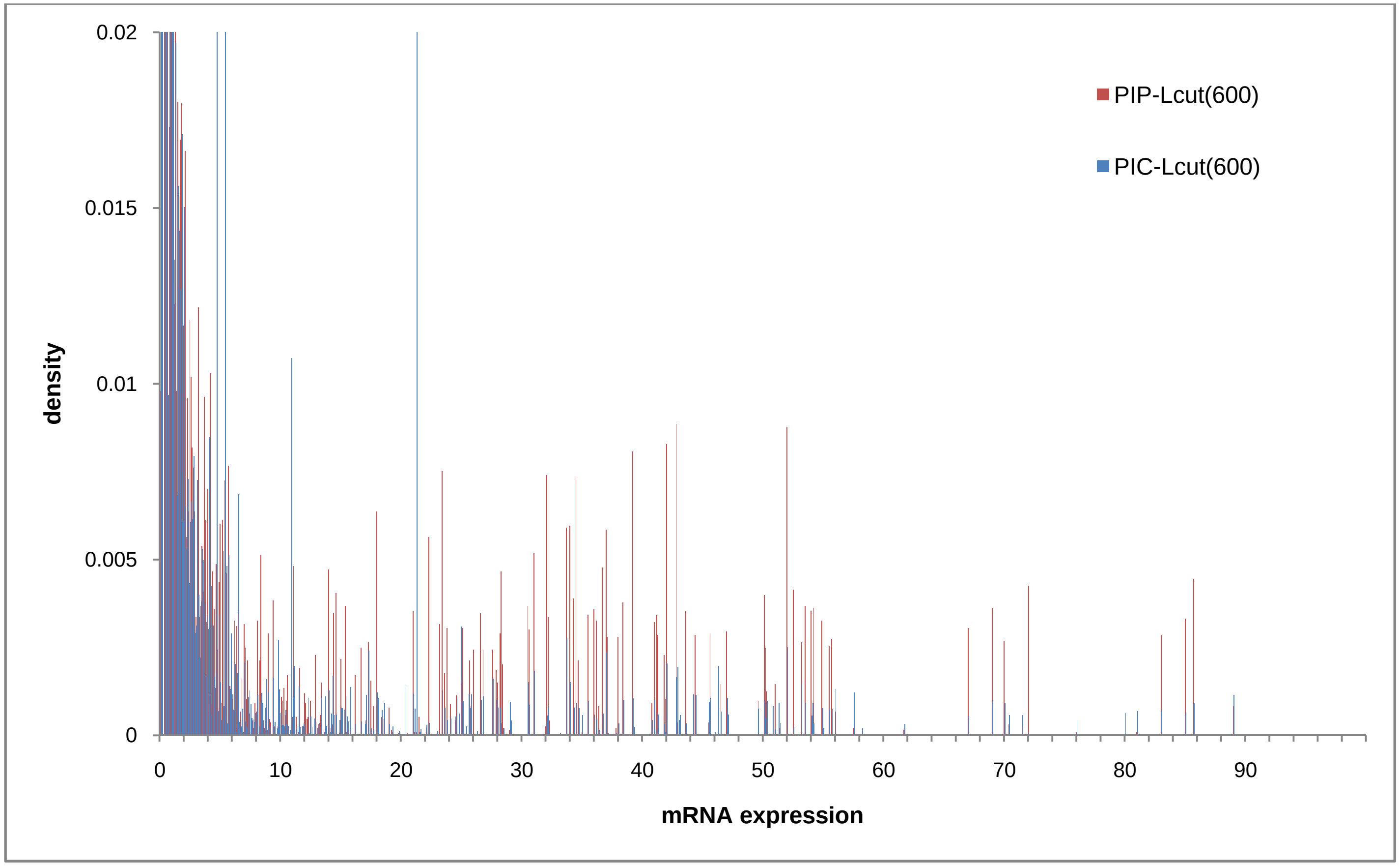


**Figure S9** Distribution of mRNA expression levels in interactions predicted by PIP-Lcut$_{600}$ (red) and PIC-Lcut$_{600}$ (blue) for *S. cerevisiae*. PIC-Lcut$_{600}$ shows less bias towards highly expressed ORFs compared to PIP-Lcut$_{600}$. Expression data are retrieved from yeast reference mRNA expression set introduced by Greenbaum *et al.* (2002, Bioinformatics 18:585-96).
