# Supplementary material for: Sequence-based prediction of protein-protein interactions by means of codon usage
Source: Genome Biol. 2008 May 23;9(5):R87. doi: 10.1186/gb-2008-9-5-r87 (PMC2441473; doi:10.1186/gb-2008-9-5-r87)
Supplement: Additional data file 10 — precision-recall curves for PIC, interolog mapping (INT), phylogenetic profiles (PGP), Rosetta stone (ROS), CAI coevolution (co-CAI) and CAI. [file gb-2008-9-5-r87-S10.pdf]

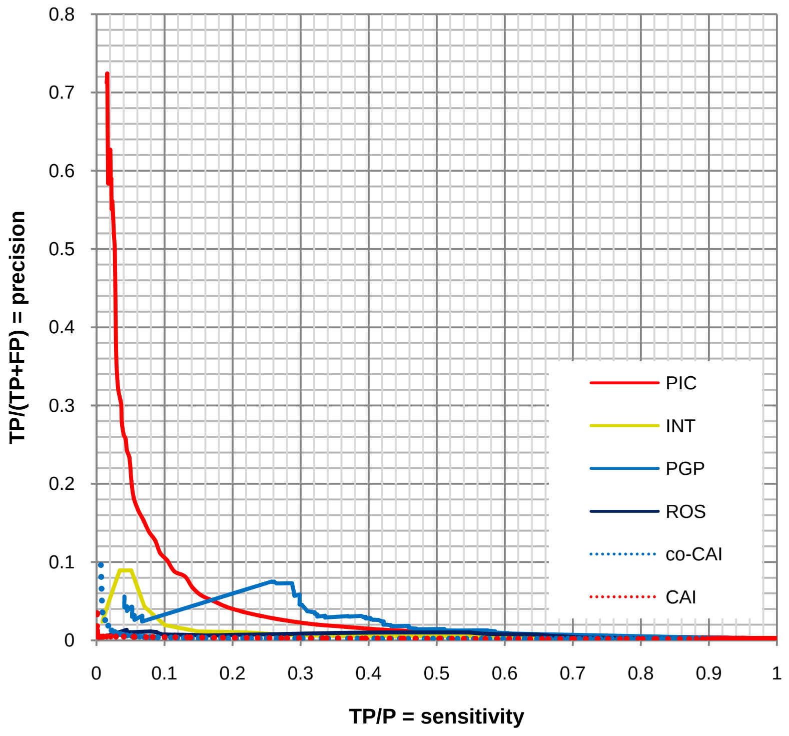

Figure S10 Comparison of precision-recall curves in yeast for PIC (red), Interolog mapping (INT, green), Phylogenetic profiles (PGP, blue), Rosetta stone (ROS, dark blue), CAI coevolution(co-CAI, blue dotted line) and absolute CAI value (CAI, red dotted line).
