# Supplementary material for: Sequence-based prediction of protein-protein interactions by means of codon usage
Source: Genome Biol. 2008 May 23;9(5):R87. doi: 10.1186/gb-2008-9-5-r87 (PMC2441473; doi:10.1186/gb-2008-9-5-r87)
Supplement: Additional data file 11 — Precision-recall curves for PIC, PIT and PICT. [file gb-2008-9-5-r87-S11.pdf]

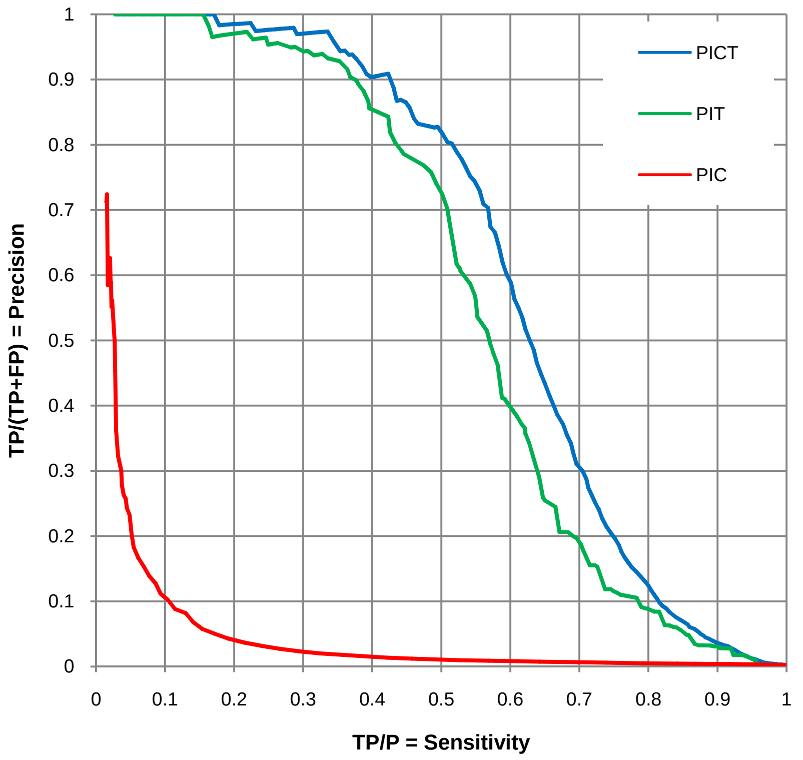

Figure S11 Comparison of precision-recall curves in yeast for PIC (red), PIT (green) and PICT (blue). At 50% precision, PICT is 12.5% more sensitive than PIT alone.
